# Management of Chronic and Neuropathic Pain with 10 kHz Spinal Cord Stimulation Technology: Summary of Findings from Preclinical and Clinical Studies

**DOI:** 10.3390/biomedicines9060644

**Published:** 2021-06-04

**Authors:** Vinicius Tieppo Francio, Keith F. Polston, Micheal T. Murphy, Jonathan M. Hagedorn, Dawood Sayed

**Affiliations:** 1 Department of Rehabilitation Medicine, The University of Kansas Medical Center, Kansas City, KS 66160, USA; kpolston@kumc.edu (K.F.P.); mmurphy11@kumc.edu (M.T.M.); 2 Department of Anesthesiology and Perioperative Medicine, Division of Pain Medicine, Mayo Clinic, Rochester, MN 55905, USA; hagedorn.jonathan@mayo.edu; 3 Department of Anesthesiology, The University of Kansas Medical Center, Kansas City, KS 66160, USA; dsayed@kumc.edu

**Keywords:** spinal cord stimulation, 10 kHz, low back pain, chronic pain, neuropathic pain

## Abstract

Since the inception of spinal cord stimulation (SCS) in 1967, the technology has evolved dramatically with important advancements in waveforms and frequencies. One such advancement is Nevro’s Senza^®^ SCS System for HF10, which received Food and Drug and Administration (FDA) approval in 2015. Low-frequency SCS works by activating large-diameter Aβ fibers in the lateral discriminatory pathway (pain location, intensity, quality) at the dorsal column (DC), creating paresthesia-based stimulation at lower-frequencies (30–120 Hz), high-amplitude (3.5–8.5 mA), and longer-duration/pulse-width (100–500 μs). In contrast, high-frequency 10 kHz SCS works with a proposed different mechanism of action that is paresthesia-free with programming at a frequency of 10,000 Hz, low amplitude (1–5 mA), and short-duration/pulse-width (30 μS). This stimulation pattern selectively activates inhibitory interneurons in the dorsal horn (DH) at low stimulation intensities, which do not activate the dorsal column fibers. This ostensibly leads to suppression of hyperexcitable wide dynamic range neurons (WDR), which are sensitized and hyperactive in chronic pain states. It has also been reported to act on the medial pathway (drives attention and pain perception), in addition to the lateral pathways. Other theories include a reversible depolarization blockade, desynchronization of neural signals, membrane integration, glial–neuronal interaction, and induced temporal summation. The body of clinical evidence regarding 10 kHz SCS treatment for chronic back pain and neuropathic pain continues to grow. There is high-quality evidence supporting its use in patients with persistent back and radicular pain, particularly after spinal surgery. High-frequency 10 kHz SCS studies have demonstrated robust statistically and clinically significant superiority in pain control, compared to paresthesia-based SCS, supported by level I clinical evidence. Yet, as the field continues to grow with the technological advancements of multiple waveforms and programming stimulation algorithms, we encourage further research to focus on the ability to modulate pain with precision and efficacy, as the field of neuromodulation continues to adapt to the modern healthcare era.

## 1. Introduction

Chronic pain is known to negatively impact patients’ social relationships, well-being, and work productivity [1,2,3,4,5,6,7,8,9,10], and is a known important risk factor for suicidality [11] and all cause-mortality [12]. Chronic low back pain is the leading cause of disability in the world, resulting in poor quality of life and limitations in daily activities [13,14]. It is the most common cause of chronic pain and the most expensive occupational disorder in the United States, leading all causes in number of work days lost annually. The impact of chronic pain on the United States healthcare system is astronomical, and the economic burden is estimated to exceed USD $500 billion per year [15]. In the United States, more than 20 million adults suffer from chronic pain of debilitating nature, and chronic pain prevalence increases with age [16,17,18,19,20,21] and continues to rise overall [19,22,23] with a 37.3% estimate in the United States and European territories. One in five of outpatient visits to health care practitioners is related to a pain complaint, which can be neuropathic (central or peripheral), nociceptive, musculoskeletal, inflammatory, or psychogenic in nature. Therefore, it is essential for the health of patients and to the health care system that clinicians have a thorough understanding of the diagnosis and management of chronic pain [24,25,26].

Diagnosing chronic pain is challenging. Patients usually present with pain and evidence of loss of function that persists beyond the expected course, usually greater than six months duration, refractory to conservative treatment, and often a focal pathoanatomical etiology cannot be found, but rather a combination of complex biopsychosocial factors is discovered. Excessive dependence on healthcare providers and family, withdrawal from social activities and work, physical deconditioning due to fear avoidance behavior, and development of psychosocial sequelae that impair function and/or recovery are identifiable patterns in patients suffering from chronic pain. When chronic low back pain is identified from a detailed history and physical examination, further evaluation with diagnostic imaging and assessment tools such as the Fear-Avoidance Behavior Questionnaire, Brief Pain Inventory, and other measures can be utilized to access the biopsychosocial complexity of chronic low back pain [24,25,26]. The management of chronic low back pain is often multidisciplinary, as it is a complex, multifactorial disease. Treatment options include exercise therapy, psychotherapy, spinal injections, surgery, and pharmacotherapy, including opioids. Chronic low back pain has a well-known association with chronic opioid therapy use with more than half of affected patients receiving at least one prescription per year. Short-term opioids may be efficacious in selected cases. However, there is limited evidence supporting long-term use, despite a continuous rise in opioid prescriptions nationwide [27,28,29].

Spinal cord stimulation (SCS) is a minimally invasive intervention utilizing electrical current to modulate pain signals that has been considered an adjunctive option in the management of chronic refractory pain. Shealy first introduced SCS implantation in 1967 based on the “gate control theory” proposed by Melzack and Wall [30,31,32]. Since its inception, the technology has evolved dramatically. One such advancement is Nevro’s Senza^®^ SCS System (Redwood City, CA, USA) for HF10, which received Food and Drug Administration (FDA) approval in 2015 and is commercially available in Europe, Australia, and the United States. Its product is an implantable high frequency spinal cord stimulation system which is thought to target the dorsal horn with HF10 therapy at 10,000 Hz or the dorsal column with lower frequency stimulation.

SCS has become a frequently performed surgical procedure for the treatment of chronic pain around the world, and now the use of SCS is considered one of the most advanced interventional methods in the treatment for chronic pain. In the United States, failed back surgery syndrome (FBSS) has been the most common reason for implantation followed by complex regional pain syndrome (CRPS), while in Europe the most common indications include intractable angina and painful peripheral vascular disease [33,34]. Multiple randomized controlled trials (RCTs) established strong evidence for use in FBSS [35,36,37,38] while economic evaluations established its long-term cost-effectiveness [39,40,41,42,43]. A recent 2019 meta-analysis of approximately 1000 patients with intractable spine and limb pain of various etiologies, including post-laminectomy syndrome, chronic back or leg pain, diabetic neuropathy, peripheral vascular disease, and CRPS, found that the utilization of SCS had better pain outcomes compared to medical treatment, with increased odds of greater than 50% pain reduction [44].

The instrumentation of SCS, as well as our understanding thereof, have been rapidly evolving with new theories, different waveforms, and programming options, in addition to emerging high-quality clinical studies discussing the role of SCS in chronic pain. Compared to conventional treatment options, SCS offers a cost-effective and reversible means for pain reduction [38].

## 2. Spinal Cord Stimulator Implantation Procedure

A spinal cord stimulator is implanted either by percutaneous or laminotomy approach. This therapy is trialed first, typically via percutaneous leads placed into the epidural space and secured to the skin, before proceeding with the surgical implant. Importantly, the surgical implant is reversible and can be removed if necessary. The implant procedure is performed in the sterile environment of an operating room with the patient prone, often using monitored anesthesia care or with general anesthesia and intraoperative neuromonitoring. Utilizing fluoroscopic guidance, the desired entry point is visualized and an incision is made just medial to the pedicle 1–2 levels below the intended interlaminar entry point. The incision is typically 1–2 inches in length. Dissection with strict hemostasis is carried until the lumbar fascia is reached and two needle introducers are directed towards the desired epidural entry point with the needle points towards the midline. Upon entering the epidural space, the SCS leads are inserted through the needles and advanced under fluoroscopy to the desired vertebral level in the dorsal epidural space. Optimal placement is confirmed fluoroscopically, and the leads are anchored to the lumbar fascia. In addition to percutaneous electrode implantation, a small laminotomy can be performed and a paddle electrode can be surgically implanted. Next, a subcutaneous pocket is created for the implantable pulse generator (IPG). The leads are tunneled to the pocket site. The incisions are copiously irrigated, hemostasis is obtained, and the leads are connected to the IPG. The SCS system is tested intra-operatively for full functionality. Lastly, the IPG is safely placed into the pocket site with any extra length of the SCS lead coiled underneath, the incisions are closed in a layered manner, and a sterile dressing is applied.

### 2.1. Advances in SCS

Historically, traditional low-frequency SCS (LF-SCS) has required maximum overlap of stimulation-induced paresthesias with painful areas to achieve pain relief [45]. As such, technological innovation had been directed at improving and enhancing the reliability of paresthesia coverage [46]. Over the last decade, however, a paradigm shift has occurred away from paresthesia mapping to paresthesia-free programming to improve patient-centered outcomes and patient satisfaction [47,48]. Several new stimulation models that elicit minimal or no paresthesias are now available, including high-frequency SCS.

### 2.2. High-Frequency 10 kHz SCS

High-frequency 10 kHz SCS works with a proposed different mechanism of action that is paresthesia-free with programming at a frequency of 10,000 Hz, low amplitude (1–5 mA) and short-duration/pulse-width (30 μS) (Table 1). High-frequency 10 kHz SCS has emerged as a superior alternative to paresthesia-based SCS and was approved for clinical use in Europe in 2011 and in the United States in 2015 [37,38].

High-frequency 10 kHz SCS offers several distinct advantages over LF-SCS. First, it applies a single waveform at 10,000 Hz frequency at a subthreshold level to provide pain relief without paresthesia, thus, eliminating the need for paresthesia overlap upon which LF-SCS relies. Second, a growing body of evidence demonstrates clinical superiority in favor of high-frequency 10 kHz SCS over LF-SCS [49,50,51,52,53]. There are numerous high-quality studies with evidence supporting the use of SCS in various conditions demonstrating superior pain relief and functional outcomes with spinal cord stimulation over comprehensive medical management, as well as opioid utilization reduction [54,55,56,57,58,59,60,61,62,63,64,65,66]. Clinical success with high-frequency 10 kHz SCS has been documented with numerous pivotal studies (Table 2).

The SENZA clinical trials highlight the extensive data regarding 10 kHz SCS, suggesting strong clinical efficacy and safety of this intervention for chronic back and lower extremity pain [52]. These studies compared 10 kHz SCS with LF-SCS in patients with back and leg pain and found a statistical and clinically significant superiority (*p* < 0.001) of high-frequency stimulation in contrast to low-frequency stimulation at six months and twenty-four months. A total of 78.7% of patients had greater than 50% back pain reduction, and similar results were reported for leg pain responders at 12-month follow-up in the 10 kHz SCS treatment arm, in contrast to 51.3% in the LF-SCS treatment arm. At 24-month follow up, 76.5% and 72.9% of patients had greater than 50% reduction in low back pain and leg pain, respectively, in the 10 kHz SCS treatment arm, compared to 49.3% in the LF-SCS treatment group. In 2020, Al-Kaisy et al. performed a sub-analysis of pooled data from the previous two prospective studies and concluded that 10 kHz SCS reduced opioid use, disability, and improved pain control in patients with non-surgical refractory back pain [55].

Over the past two decades, in summary, research studies have demonstrated the clinical and statistical efficacy of high-frequency 10 kHz SCS in patients with chronic refractory low back pain with neuropathic pain and post-laminectomy syndrome [49,68], in addition to painful diabetic neuropathy [64,65,69], non-surgical refractory back pain [55,70,71], chronic neck pain [57,58,62,72,73,74], chronic regional pain syndrome (CRPS) [75,76,77,78,79], thoracic pain [67], chronic post-surgical pain [66], chronic pelvic pain [63,80], chronic abdominal pain [81], migraine [82,83], as an adjunctive treatment do decrease opioid utilization [54,59,84], and as salvage therapy [85,86]. Among other painful syndromes in which 10 kHz SCS has been studied, painful diabetic neuropathy (PDN) and chronic neck and upper extremity pain have continued to gain clinical and research interest, as well as the use of 10 kHz SCS as a salvage therapy. More recently, multicenter studies by Petersen et al. and Galan et al. reported the outcomes of 10 kHz SCS in PDN and found an overall 74% pain relief which was sustained at 12 months with a total of 86% of subjects being responders and up to 71% of subjects reporting improvement in peripheral sensory and reflexes [64,65]. The retrospective open-label study by El-Majdoub et al. [72] evaluated the efficacy of 10 kHz in chronic neck and upper limb pain, and the authors reported 71.6% pain relief at three months, 70.9% at six months, and 74.1% at 12 months with concomitant improvements in upper limb pain and the Oswestry Disability Index (ODI). Amirdelfan et al. reproduced similar positive outcomes in a prospective design with 10 kHz SCS, reporting 89.2% of subjects with neck pain and 95% of subjects with upper limb pain had at least 50% pain relief from baseline at 12-month follow-up [57]. Recently, Kapural et al. conducted a retrospective data review of two clinical sites to access the utilization of 10 kHz SCS as a salvage therapy and found that 81% of the cases reviewed reported at least 50% pain relief upon utilization of 10 kHz SCS as salvage therapy with significantly decrease in opioid utilization at 12 months post-procedure [85].

In sum, among the published literature, there appears to be significant favorability towards high-frequency 10 kHz SCS in contrast to low-frequency SCS (Table 2). With improved efficacy, higher responder rates, and a more favorable risk profile as compared with alternative treatments, high-frequency 10 kHz SCS is poised to impact the management of multiple chronic pain conditions for the foreseeable future.

Despite the clinical success of 10 kHz SCS, the underlying mechanism of action has yet to be fully elucidated. Just as the electrical characteristics between high-frequency 10 kHz SCS and LF-SCS differ, so too do the mechanisms of action [87,88]. Low-frequency SCS activates large-diameter pain fibers at the segmental level to produce paresthesia. In keeping with segmental concepts, high-frequency 10 kHz SCS is thought to instead target local dorsal horn neurons more directly, without activation of dorsal column fibers [89]. In addition to these segmental effects, several other hypotheses including supraspinal mechanisms have been posited to contribute to the overall effectiveness of 10 kHz SCS.

Until recently, there has been a relative gap in the existing literature as it pertains to the mechanisms of action for 10 kHz SCS. The aim of this review is to describe the current literature in order to identify the specific therapeutic mechanisms at play in high-frequency 10 kHz SCS. A clearer understanding of 10 kHz SCS mechanisms will help delineate which patients may benefit from which waveforms and thus allow for individual optimization of neural and segmental features to treat pain. The following sections discuss the mechanisms through which pain relief is thought to be achieved by SCS and high-frequency 10 kHz SCS.

## 3. Paresthesia Based Therapy and Its Mechanism (Gate Control Hypothesis)

It has previously been suggested that the analgesic mechanism for LF-SCS occurs via the gate control theory through a tonic stimulation pattern applied with a frequency ranging from 20 to 120 Hz and a pulse width varying between 200 and 500 µs. This tonic stimulation waveform delivers adjustable amplitudes based on the patient’s subjective sensation of paresthesia and induced pain relief. Ideally, the perception of paresthesia should be between the perception threshold and the discomfort threshold, also known as the therapeutic window of amplitude stimulation. Pulse width can be adjusted to widen or narrow the electrical field and frequency can be adjusted to alter the perceived sensation. This hypothesis relies on the notion that activation of myelinated large diameter A-beta fibers traveling orthodromically to the thalamus before reaching the cortex inhibits transmission of nociceptive information at the segmental level [31,89,90,91]. Yang et al. showed that a positive correlation between stimulation intensity and degree of inhibition of wide dynamic range (WDR) neurons exists [92]. Clinically, the result is paresthesia, which is often perceived by patients as numbness, tingling, pressure, pins and needles, or buzzing. Thus, pain relief is achieved by overlapping areas of paresthesia with areas of pain (paresthesia mapping).

However, this gate control theory alone does not fully explain the mechanistically significant role high-frequency 10 kHz SCS plays in pain relief. Additional mechanistic theories that will be explored in this review include: (1) Segmental, antidromic activation of A-beta efferent neurons, (2) Blocked transmission in the spinothalamic tract, (3) Supraspinal pain inhibition, (4) Activation of central inhibitory mechanisms influencing sympathetic efferent neurons, and (5) Activation of putative neurotransmitters or neuromodulators.

## 4. Studies Supporting the Efficacy of the 10 kHz SCS Mechanism

### 4.1. In Vitro/Ex Vivo Model

High-frequency 10 kHz SCS has been shown to alleviate pain without the undesired effect of paresthesia. This is in stark comparison to traditional high-intensity, low-frequency (<100 Hz) SCS, which preferentially activates interneurons in the dorsal horn to produce paresthesia-based pain relief. Lee et al. sought to investigate the paresthesia-free analgesic mechanism of high-frequency 10 kHz SCS by performing in vivo and ex vivo electrophysiological experiments (Table 3) [91]. They used in vivo animal recordings, corroborated by ex-vivo recordings of GABAergic inhibitory neurons, to explore the ability of 10 kHz SCS to modulate spinal dorsal horn neuronal function. They found that the firing rates of non-adapting cells (inhibitory) and adapting cells (excitatory) at 30% (sub-sensory threshold), as well as 60% and 90% (above sensory threshold) motor thresholds (MT) differed. Specifically, 10 kHz SCS selectively activated inhibitory interneurons in the dorsal horn at 30% of MT but activated both inhibitory and excitatory interneurons in the dorsal horn at 60% and 90% MT. Furthermore, 1 kHz and 5 kHz SCS at 30% MT did not demonstrate a significant increase in non-adapting cell firing rate [91]. Thus, it appears likely that low-intensity (sub-sensory threshold) 10 kHz SCS effectively provides paresthesia-free pain relief by preferentially activating putative inhibitory interneurons without activating excitatory interneurons in the dorsal horn. It remains unclear whether this mechanism is selective to pain modulation as opposed to having alternative effects within the spinal cord (i.e., being involved in segmental sensory-motor transmission). Nonetheless, the ability to modulate pain through activation of inhibitory interneurons without triggering paresthesia-mediated excitatory neurons is a significant advantage of high-frequency 10 kHz SCS over low-frequency SCS.

### 4.2. In Vivo/Animal Model

Additional animal studies have begun to elucidate the biochemical and neuromodulatory bases by which high-frequency 10 kHz SCS ameliorates chronic pain. Mitogen-activated protein kinases (MAPKs) are thought to play an important role in neural plasticity and inflammatory response following painful stimuli. Three MAPK proteins that have been established as important mediators of these responses with effects on hypersensitivity, nociception, and allodynia include extracellular signal-related protein kinases (ERKs), p38, and c-Jun N-terminal kinases (JNKs) [98,99,100]. Following nerve injury, these MAPKs are activated (phosphorylated), leading to prolonged pain, nociception, and hypersensitivity. Furthermore, inhibition of these MAPK pathways has been shown to attenuate pain in previous animal models [101]. As such, these pathways represent a potential mechanism by which 10 kHz SCS may mitigate pain without producing paresthesia. Liao et al. sought to examine this mechanism by evaluating phosphorylation of MAPKs in response to pain to elucidate a potential underlying mechanism for pain relief produced by high-frequency 10 kHz SCS [93]. This study demonstrated that animals with high-frequency 10 kHz SCS exhibit significantly lower levels of phosphorylation of ERK1, ERK2, JNK1, and p38 in the dorsal root ganglion and dorsal horn, suggesting a possible molecular basis by which 10 kHz SCS ameliorates pain without producing paresthesia. Differences in phosphorylation in this protein signaling network were noted between HFSCS and non-HFSCS groups as early as one day following induced spinal nerve injury, suggesting that modulation of MAPK proteins is an early mechanism of 10 kHz SCS.

In addition to MAPKs, glutamate receptors have been studied in the molecular pathogenesis of neuropathic pain and pain secondary to nerve injury. The process of central sensitization following nerve injury involves neuroplastic changes in response to nociceptive stimuli, with a key component of this plastic process being changes in glutamate receptors [102]. Accordingly, change in glutamatergic signaling is a mechanism by which 10 kHz SCS has been postulated to potentially impact chronic pain pathways. Liao et al. found that high-frequency 10 kHz SCS in rats with spared nerve injury (SNI)-induced neuropathic pain led to attenuation of the increases in spinal glutamate release [94]. This study examined five groups (*n* = 10 in each group) of animals: a naive control group, a sham group with SCS lead implantation without electrical stimulation, a sham group with high-frequency 10 kHz SCS, an SNI group with lead implantation without electrical stimulation, and an SNI group with high-frequency 10 kHz SCS. The animals were tested for SNI-induced mechanical and cold allodynia, cerebrospinal fluid (CSF) levels of glutamate, glutamate transporter activity, and miniature excitatory postsynaptic current (mEPSC) transmission. High-frequency 10 kHz SCS treatment in rats was found to decrease both mechanical and cold allodynia in SNI-induced neuropathic pain. Additionally, spinal CSF levels of glutamate were found to be increased following sciatic nerve injury, and these increases were reversed by treatment with 10 kHz SCS. Furthermore, high-frequency 10 kHz SCS treatment resulted in increased glutamate transporter activity following SNI, but did not result in changes in expression of glutamate transporter. Miniature excitatory postsynaptic currents in lamina II neurons were found to be increased following SNI regardless of treatment group. Finally, 2-methyl-6-(phenylethynyl) pyridine (MPEP), a metabotropic glutamate 5 receptor (mGluR5) allosteric inhibitor, and (RS)-2-Chloro-5-hydroxyphenylglycine (CHPG), a mGluR5 agonist, were injected intrathecally and resulted in significant alterations to 10 kHz SCS induced analgesia. Injection of MPEP resulted in an antinociceptive effect on allodynia, while CHPG resulted in a pronociceptive effect on allodynia.

These studies examining changes in neurotransmitters in response to high-frequency 10 kHz SCS begin to elucidate the biochemical basis by which it produces pain relief without producing paresthesias and furthermore provides evidence to support its use to modulate the neurochemical process leading to pain.

### 4.3. Functional MRI (MRI) in Chronic Pain Patients

Human studies have begun to elucidate the anatomical mechanisms by which high-frequency 10 kHz SCS affects the experience of pain. De Groote et al. examined these mechanisms using resting state functional magnetic resonance imaging (rsfMRI) [95]. In this study, ten individuals with failed back surgery syndrome underwent rsfMRI at baseline and after 1- and 3-month intervals after 10 kHz SCS. The authors found that over time, connectivity increases between the anterior insula and regions from the frontoparietal network (the lateral prefrontal cortex and inferior parietal cortex). This study suggests that the anterior insula may play a role in modifying the pain response and may serve as an integrator for pain relief in these patients.

Additionally, high-frequency 10 kHz SCS appears to lead to changes in volume of specific brain regions [96]. De Groote et al. analyzed hippocampal volumes in 11 individuals with failed back surgery syndrome who underwent HF-SCS implantation. Magnetic resonance imaging was obtained at baseline as well as after 1- and 3-month intervals after HF-SCS placement. Patients in the study experienced a significant decrease in numeric rating scale (NRS) score for pain intensity. Additionally, MRI measurements using automated voxel-based morphometry found significantly decreased bilateral hippocampal volumes following three months of high-frequency 10 kHz SCS (but no significant difference after only one month). Furthermore, decreases in NRS scores were found to significantly correlate with the observed decreases in hippocampal volume.

These studies demonstrate that high-frequency 10 kHz SCS modulates the experience of pain not only at the neurochemical level, but also at the neuroanatomical level, leading to changes in activity in areas of the brain associated with chronic pain. The ability to modulate or modify these anatomical pathways may serve as a basis for understanding why high-frequency 10 kHz SCS produces superior pain relief versus LF-SCS in many patients.

### 4.4. 10-Channel EEG in Chronic Pain Patients

Studies have also begun to use 10-channel electroencephalogram (EEG) to analyze the differences in neural patterns generated when using 10 kHz SCS versus 60 Hz tonic SCS. Telkes et al. investigated these differences in nine patients undergoing SCS implantation [97]. This study found that individuals undergoing high-frequency 10 kHz SCS implantation experienced stronger alpha wave power in the somatosensory region of the brain than individuals undergoing LF-SCS implantation. Furthermore, high-frequency 10 kHz SCS individuals were found to have a shift in peak frequency from theta to alpha waves as compared to their baseline and compared to the LF-SCS group. In addition, there was a positive correlation between Oswestry disability index (ODI) scores and the relative increase in alpha waves in frontal and somatosensory regions in high-frequency 10 kHz SCS. These findings demonstrate early electrophysiological changes that may elucidate the response to SCS therapy prior to device selection, potentially reducing health care expenses related to implant failure, in addition to provide insight into the effects of SCS therapy on brain activity and subconscious pain mechanisms.

## 5. Summary

High-frequency 10 kHz SCS involves a paresthesia-free paradigm as stimulation occurs below the sensory threshold, in contrast to LF-SCS, which relies on paresthesia production and pain overlap. Several working hypotheses for the mechanisms of pain relief with 10 kHz SCS have been proposed [103,104,105,106,107,108], including a reversible depolarization blockade (limiting the propagation of nociceptive signals), desynchronization of neural signals (resulting in pseudo-spontaneous or stochastic neuronal activity in the spinal gate), membrane integration, glial-neuronal interaction, and induced temporal summation, which attenuates the WDR wind-up phenomenon, representing suppression of hyperexcitability in spinal cord neurons. We believe the mechanism of action based on theoretical hypotheses and computational modeling need to be supported by findings from in vitro/in vivo/ex vivo studies. Therefore, preclinical and clinical studies documenting cellular/tissue level events following 10 kHz SCS treatment were discussed in the review.

The body of clinical evidence regarding high-frequency 10 kHz SCS treatment for chronic pain continues to grow as the technology in spinal neuromodulation evolves. There is high-quality evidence supporting is use in patients with persistent back and radicular pain, particularly after failed spinal surgery. Kapural et al. published a multicenter randomized clinical trial of patients with heterogeneous diagnoses, and compared high-frequency 10 kHz SCS therapy with LF-SCS. Data suggested that the HF-SCS group had a greater response rate relative to LF-SCS and greater pain reduction in both back and leg regions at 24-month follow-up (8). The SENZA series of trials introduced robust data regarding the use of high-frequency 10 kHz SCS and provides substantial evidence that √ is not only safe, but also statistically and clinically effective with demonstrated superiority of high-frequency 10 kHz SCS in contrast to LF-SCS in the treatment of chronic back and neuropathic pain, with level I evidence supported by real-world data studies and clinical experience [67,68,70,84,109,110,111].

## 6. Future Perspectives

Our understanding of the 10 kHz SCS MOA has expanded, and there is high-level, well-established evidence that high-frequency 10 kHz SCS is a safe, minimally invasive, cost-effective treatment option with high patient satisfaction in multiple chronic pain conditions. High-frequency 10 kHz SCS has been shown to improve pain at short- and long-term follow-up, reduce disability, improve functional scores, decrease opioid utilization, and improve quality of life; therefore, it is feasible to consider earlier use of this intervention in the chronic pain treatment algorithm.

The future of SCS is bright with significant technological advancements in waveforms and programming algorithms, in addition to the creation of devices with the ability to deliver multiple stimulation settings. The expanded options offer the ability to modulate pain with precision and efficacy in a variety of ways, as the field of neuromodulation continues to adapt in the modern healthcare era. As the field continues to grow, we encourage further research regarding potential changes in pain biomarkers with the use of 10 kHz SCS and additional applications of this therapy in other chronic pain conditions and spinal cord diseases, such as spasticity and motor control after spinal cord injury.

## Figures and Tables

**Table 1:** Characteristics of 10 kHz SCS.

Frequency	10,000 Hz
Amplitude	1 to 5 mA (low-amplitude)
Pulse width	short-duration (30 s)
Typical lead placement for back and/or leg pain	distal tip of one lead is placed at T8 and a second lead tip is placed at T9 both near the anatomical midline (based on extensive empirical observations).
FDA approval status	Approved in 2015 for treatment of chronic pain in trunk and limbs.
Follow-up data available for patients	12 months
	24 months

**Table 2:** Pivotal studies documenting the efficacy of 10 kHz SCS.

Study (First Author et al., Year of Publication)	Pain Type/Area	Study Design, *n*, Follow-Up	Pain Relief and Responder (≥50% Pain Relief) Rate at Last Follow-Up
Al-Kaisy et al., 2014 [49]	Chronic back and leg pain	Single arm, prospective *n* = 65 Follow-up, 24 months	Average pain relief at 24 months: back pain, 61%; leg pain, 57%. Responder rate at 24 months: back pain, 60%; leg pain 71%
Kapural et al., 2016 [52]	Chronic back and leg pain (predominant back pain)	Prospective, RCT *n* = 85 in 10 kHz SCS group Follow-up, 24 months	Average pain relief baseline to 24 months: back pain 68%; leg pain 66% Responder rate at 24 months: back pain 77%; leg pain 73%
Stauss et al., 2019 [56]	Chronic pain in trunk and limbs	Single-arm, retrospective *n* = 1661 Mean follow-up time, 8.9 months (range: 0.1 to 33.2 months)	Median pain relief (VRS) at last visit: 62% Responder rate at last visit: 74%.
Kallewaard et al., 2020 [61]	FBSS (predominant leg pain)	Single arm, prospective *n* = 50 Follow-up, 12 months	Average pain relief baseline to 12 months: back pain, 63%; leg pain, 75%. Responder rate at 12 months: back pain, 76%; leg pain, 80%.
Amirdelfan et al., 2020 [57]	Chronic upper limb and neck pain	Single arm, prospective *n*= 45 Follow-up, 12 months	Average pain relief baseline to 12 months: upper limb pain, 86%; neck pain, 79%. Responder rate at 12 months: upper limb pain, 95%; neck pain by 89%.
Sayed et al., 2020 [62]	Chronic upper limb and neck pain	Single arm, retrospective *n* = 47 Median follow-up time, 19.4 months	Average pain relief baseline to last follow up: 58% Responder rate at last follow up: 76%
Sayed et al., 2020 [67]	Thoracic back pain	Single arm, retrospective *n*= 19 Follow-up, 12 months	Average pain relief baseline to 12 months: 70% Responder rate at 12 months: 89%
Tate et al., 2021 [63]	Chronic pelvic pain	Single arm, prospective *n* = 13 Follow-up, 12 months	Average pain relief baseline to 12 months: 72% reduction. Responder rate at 12 months: 77%
Gupta et al., 2020 [66]	Chronic post-surgical pain	Single arm, prospective *n*= 25 Follow-up, 12 months	Average pain relief baseline to 12 months: 82% reduction Responder rate at 12 months: 88%
Peterson et al., 2021 [65]	Painful diabetic neuropathy	RCT *n* = 87 in 10 kHz SCS group Follow-up, 6 month assessment reported	>Average pain relief baseline to 6 months: Lower limb: 78% Responder rate at 6 months: 85%

**Table 3:** Studies documenting MoA of 10 kHz SCS.

Study (First Author et al., Year of Publication)	Type (In Vitro, Ex Vivo, In Vivo or Clinical)	Model	Key Finding
Lee et al., 2020 [91]	In vivo and ex vivo	In vivo and ex vivo electrophysiological approaches: In vivo experiments: adult male Sprague Dawley rats Ex vivo experiments: Transgenic mice expressing green fluorescent protein in GABAergic neurons	10 kHz SCS may inhibit pain sensory processing in the spinal dorsal horn by uniquely activating inhibitory interneurons without activating dorsal column fibers, resulting in paresthesia-free pain relief.
Liao et al., 2020 [93]	In vivo, sham controlled	Spared nerve injury Sprague-Dawley rats	10 kHz SCS applied to the T10/T11 spinal cord significantly attenuated spared nerve injury-induced mechanical hyperalgesia compared with the sham stimulation group. Western blotting revealed a significant attenuation of ERK1, ERK2, JNK1, and p38 activation in the dorsal root ganglia and the spinal dorsal horn.
Liao et al., 2020 [94]	In vivo, sham controlled	Spared nerve injury Sprague-Dawley rats	10 kHz SCS treatment attenuated spared nerve injury -induced neuropathic pain and partially restored the altered glutamate uptake after spared nerve injury.
DeGroote et al., 2020 [95]	Clinical, Prospective study	Patients with FBSS treated with 10 kHz SCS; resting state functional magnetic resonance imaging (rsfMRI)	Increased strength in functional connectivity between the left dorsolateral prefrontal cortex and the right anterior insula significantly correlated with the minimum clinically important difference value of the Pittsburgh sleep quality index.
DeGroote et al., 2020 [96]	Clinical, prospective	Patients with FBSS treated with 10 kHz SCS; neuroimaging MRI (Voxel-Based Morphometry Diffeomorphic Anatomical Registration Through Exponentiated Lie)	10 kHz SCS influences structural brain regions over time. The volume of the hippocampus decreased bilaterally after three months with a positive correlation with back pain intensity.
Telkes et al., 2020 [97]	Clinical, prospective	Patients with FBSS treated with 10 kHz SCS; electroencephalogram (EEG)	Stronger relative alpha power in the somatosensory region. Shift in peak frequency from theta to alpha Rhythms compared to baseline. Changes in ODI scores positively correlated with alpha/theta peak power ratio in frontal and somatosensory regions.

## Data Availability

No new data were created or analyzed in this study. Data sharing is not applicable to this article.
